# Development of Diagnostic Tests for Detection of SARS-CoV-2

**DOI:** 10.3390/diagnostics10110905

**Published:** 2020-11-05

**Authors:** Ngan N. T. Nguyen, Colleen McCarthy, Darlin Lantigua, Gulden Camci-Unal

**Affiliations:** 1 Department of Chemical Engineering, University of Massachusetts Lowell, One University Avenue, Lowell, MA 01854, USA; Ngan_Nguyen@student.uml.edu (N.N.T.N.); Colleen_McCarthy1@student.uml.edu (C.M.); Darlin_Lantigua@student.uml.edu (D.L.); 2 Biomedical Engineering and Biotechnology Program, University of Massachusetts Lowell, One University Avenue, Lowell, MA 01854, USA; 3 Department of Surgery, University of Massachusetts Medical School, 55 Lake Avenue, Worcester, MA 01655, USA

**Keywords:** COVID-19, SARS-CoV-2, diagnostic tests, viral infections, disease detection

## Abstract

One of the most effective ways to prevent the spread of the severe acute respiratory syndrome coronavirus-2 (SARS-CoV-2) is to develop accurate and rapid diagnostic tests. There are a number of molecular, serological, and imaging methods that are used to diagnose this infection in hospitals and clinical settings. The purpose of this review paper is to present the available approaches for detecting SARS-CoV-2 and address the advantages and limitations of each detection method. This work includes studies from recent literature publications along with information from the manufacturer’s manuals of commercially available SARS-CoV-2 diagnostic products. Furthermore, supplementary information from the Food & Drug Administration (FDA), Centers for Disease Control and Prevention (CDC), and World Health Organization (WHO) is cited. The viral components targeted for virus detection, the principles of each diagnostic technique, and the detection efficiency of each approach are discussed. The potential of using diagnostic tests that were originally developed for previous epidemic viruses is also presented.

## 1. Introduction

An outbreak of a new pathogenic virus, the severe acute respiratory syndrome coronavirus-2 (SARS-CoV-2), was identified in China in mid-December 2019 and spread globally [1]. This new virus, which has a similar structure to the virus related to the SARS epidemic in 2003, was identified from a bat species. In a previous phylogenetic study by Mackenzie et al. (2020), the virus associated with the 2019 coronavirus disease (COVID-19) pandemic shares approximately 80% of its nucleotide genome with SARS-CoV [2]. Specifically, two previous SARS-CoV viruses, one identified from Chinese horseshoe bats (Rhinolophus sinicus) in Zhoushan and the other identified from an Intermediate horseshoe bat (R.affinis), shared 89% and 96% of their nucleotide genomes, respectively, with SARS-CoV-2 [2,3,4]. Due to the similarity between the two viruses, the 2019-nCoV virus was named SARS-CoV-2. Even though, the intermediate host has not been confirmed, several possible hosts include snakes, pangolins, turtles, or other wild animals [5].

According to a WHO report, this new virus is transmitted through respiratory droplets or close contact of patients with COVID-19 [6]. As of 30 September 2020, there have been 48 million confirmed cases of COVID-19 around the world [7]. On the same date, CDC databases reported that there had been 9,268,818 confirmed cases and 230,893 deaths in the United States [8]. Governments around the world have established social distancing requirements, travel restrictions, and mandatory quarantines to prevent the spread of the virus. Despite these efforts, the virus has continued to spread, and the pandemic has severely impacted the living and working conditions of people across the globe. A large number of people have lost their jobs, and incidents of domestic violence, such as physical, emotional, and sexual abuse, have rapidly increased. According to the US Bureau of Labor Statistics, the unemployment rate in September 2020 was 7.9%. This rate is significantly higher than the rates observed over the last five years, which ranged from 3.5% to 5.5% [9]. Based on data recorded by domestic abuse charities in the UK, the number of emergency calls related to domestic abuse have increased by 25% since the lockdown announcement [10]. Many businesses throughout the world economy, including those in the electronic, travel, and financial industries have been shut down or heavily restricted [11]. Therefore, to ease the severity on social and economic impacts as a result of the COVID-19 pandemic, efforts should be put in the development of rapid and reliable technologies to timely detect and reduce the spread of the virus to ease our way back to normality.

Some of the challenges associated with the detection of SARS-CoV-2 include the abundance of asymptomatic cases and the difficulty of symptom recognition. Asymptomatic carriers, who may not recognize that they are infected can still spread the virus by close contact with other people. According to a report by Mizumoto et al., 18% of the positive cases on the Diamond Princess cruise ship were identified as asymptomatic [12]. The basic reproductive number (R0) for the cruise ship outbreak was 4 times higher than the productive number observed in Wuhan [12,13]. In another report on 217 passengers on an Argentinian expedition cruise ship, 57% of passengers tested positive for COVID-19. Of the passengers with the virus, 81% were asymptomatic [14]. These statistics suggest that the actual number of people who have contracted the SARS-CoV-2 virus might be significantly higher than the number that has been reported.

The second challenge in detecting SARS-CoV-2 is the difficulty of identifying and recognizing the symptoms. In the early stages of the COVID-19 outbreak, the virus was detected by looking for symptoms such as fever, dry cough, and shortness of breath. In 2020, Wang et al. studied 138 novel coronavirus-infected pneumonia (NCIP) patients in a Wuhan hospital [15]. The study found that 98.6% patients experienced fever, 69.6% patients experienced fatigue, and 59.4% of patients experienced a dry cough [15]. These symptoms can be the same as those for other respiratory illnesses such as the common cold, influenza, and seasonal allergies. The similarity of symptoms between COVID-19 and other respiratory illnesses makes it difficult to recognize a SARS-CoV-2 infection. Moreover, the incubation period of the virus varies greatly between people. As a result, many patients may not recognize that they are infected for some time. Based on a CDC report, the symptoms of an infected person usually appear 5 days after infection occurs [16]. It is possible, however, for symptoms to appear anytime between 3 and 14 days after infection [16]. Due to these issues, the development of efficient, rapid, and reliable tests for mass testing of populations can improve early detection and significantly decrease further spread of the virus.

The SARS-CoV-2 detection techniques currently used by advanced laboratories and commercial companies fall into three categories: (i) Molecular methods capable of detecting viral RNA sequences, (ii) rapid diagnostic tests (RDT) that can detect the presence of the virus based on antigens or host antibodies, and (iii) imaging techniques that can detect changes in the lung. In the molecular approach, polymerase chain reaction (PCR)-based and high-throughput sequencing technologies are commonly used to replicate nucleic acids and detect the presence of the virus in respiratory samples [17]. In RDT tests, either antigens or antibodies are used to detect the presence of the virus. For antibody-based testing, ELISA or immune-based technologies are used to detect antibodies in patient samples and determine whether a patient was previously infected. Antibody tests are performed using blood, plasma, or serum samples. Antigen-based testing methods can detect the presence of viral antigens in respiratory samples and diagnose an active infection. Lastly, radio-imaging methods, such as computed tomography (CT), are used to monitor the shape of the chest over the course of an infection. Table 1 presents various testing methods and evaluates them based on the testing materials, detection platforms, detection targets, and limits of detection. These methods are also evaluated for sensitivity, specificity, and accuracy in Table 2. Table 3 summarizes the currently available antigen-based tests for SARS-CoV-2. This review will discuss the principles, advantages, and disadvantages of each approach. Additionally, this paper will discuss detection approaches that were used for previous epidemic viruses to demonstrate the possibility of their use in the detection of SARS-CoV-2.

## 2. Components of SARS-CoV-2 Used for Detection

**Figure 1:**
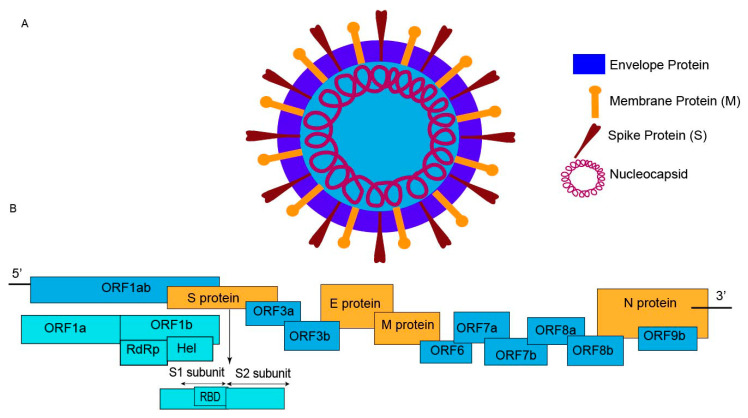
(**A**) The schematic of the SARS-CoV-19 virus displays a spherical structure. It contains spike surface (S) proteins, envelope (E) proteins, and matrix membrane (M) proteins on the outer surface. The association between single-stranded RNA and nucleocapsid proteins is included in the inner surface. (**B**) The genomic structure of SARS-CoV-2 is shown from the 5′ end to the 3′ end. The open reading frame (ORF)1ab gene, S gene, and N gene are typically used for nucleic acid testing. Some laboratories and commercial companies target RdRp and Hel enzymes. S proteins and N proteins are typical targets for serological COVID-19 tests.

The SARS-CoV-2 virus, which belongs to the beta-coronavirus group, is described as a large, enveloped virus with a genome made up of positive, single-stranded RNA molecules. The virus can infect humans and other animals, and the size of its genome is approximately 29.9 kb [17]. A schematic diagram of the SARS-CoV-2 virus is shown in Figure 1.

### 2.1. Non-Structural Proteins

The genome of SARS-CoV-2 includes a variable number of open reading frames (ORFs) starting at the 5′-end. In the first ORF, more than two-thirds of the genetic sequence encodes 16 non-structural proteins (nsps). The remaining ORFs encode accessory proteins (HE, 3a/b, and 4a/b proteins) and structural proteins [27,28]. In the ORF1ab region, the RNA-dependent RNA polymerase (RdRp) enzyme, which is indispensable during genome replication, conjugates with nsps to maintain the accuracy of the genome [28,29]. During the synthesis of viral RNA, the ORFs regions (RdRp, Hel) can be used as catalysts to improve the replication and transcription cycles of SARS-CoV-2. Therefore, these ORFs regions have become potential targets for the detection of SARS-CoV-2 with nucleic acid testing methods.

### 2.2. Structural Proteins

The β-coronavirus contains four structural proteins: Spike surface glycoproteins (S), small envelope proteins (E), matrix membrane proteins (M), and nucleocapsid proteins (N). S proteins, which are located at the outer surface of the virus, contain three segments: A large ectodomain, a single-pass transmembrane anchor, and a short intracellular tail. These ectodomains are divided further into two domains: The N-terminal domain (S1) and the C-terminal domain (S2) [30]. S proteins enable the virus to enter the host cells. Human angiotensin-converting enzyme 2 (ACE2), which is found on the surfaces of many cell types, is a host receptor for SARS-CoV-2. Human ACE2 plays one of the most significant roles in viral entry. The S proteins bind to a host receptor through the receptor-binding domain (RBD) in the S1 subunit and fuse the viral and host membranes through the S2 subunit [31]. As a result, S proteins can be potential targets for detecting the presence of SARS-CoV-2. The spike proteins on the SARS-CoV-2 virus are very similar to those found on the SARS-CoV virus. According to a report completed by Wang et al., the spike proteins of SARS-CoV shared 92% of their identity with the SARS-CoV-2 spike proteins [32]. Therefore, interference testing needs to be completed when using S proteins as the detection targets.

N proteins are critical for early viral replication in the host cells. N proteins play a primary role in packaging viral RNA into ribonucleoprotein (RNP) complexes called nucleocapsids [33]. When the virus enters the host cells, nucleocapsid proteins support the replication of viral RNA and release virus particles into the host cells [34,35]. Due to the importance of N proteins for viral replication, they can be used in the development of diagnostic tests for SARS-CoV-2. Based on the location and function of these proteins, both S and N proteins have become potential targets for detecting SARS-CoV-2 related antigens and antibodies. Additionally, S and N proteins can be helpful for identifying changes in the virus’ genome sequence over the course of an infection.

### 2.3. Antibodies

An antibody or immunoglobin (Ig), which is considered a protective protein, is produced as part of an immune response to an antigen. One method that can be used to determine a COVID-19 infection, is the detection of antibodies (IgM, IgG, and IgA) in the body. IgM, which accounts for approximately 10% of human immunoglobin, is the first antibody produced by B cells after infection occurs [36]. It is also the first antibody that is secreted to the blood stream during an immune response [37]. IgM has a pentameric structure with five Y-shaped molecules and a high affinity for specific antigens [36]. IgG is the second antibody produced during an immune response. It accounts for approximately 70–75% of human immunoglobin found in the body and plays a significant role in developing long-term immunity [36]. IgA, which accounts for 10%–15% of human immunoglobin, can be found in serum, nasal mucus, saliva, breast milk, etc. [36]. Both IgG and IgM are used to determine whether a person is currently infected or was previously infected by SARS-CoV-2. These immunoglobins are used because they are the first antibodies produced and are found in large quantities in the human body. IgA can also be used as a target for the detection of SARS-CoV-2, but its use is less common [38,39].

### 2.4. Interleukin-6

Pleotropic cytokine, commonly known as interleukin-6 (IL-6), plays a significant role in innate and adaptive immunity [40]. It is produced after a SARS-CoV-2 infection as a result of damage to stromal cells and immune system cells (B lymphocytes, T lymphocytes, macrophages, monocytes) [41,42]. IL-6 modulates the host immune response and indicates the presence of extreme inflammation [40]. The concentration of IL-6 in the blood can provide insight into the severity of sepsis, septicemia, and infection in patients who require intubation and mechanical ventilation [43,44,45]. According to a study by Herold et al., respiratory failure was 22 times more severe when COVID-19 patients had IL-6 levels greater than 80 pg/mL [44]. Therefore, IL-6 is an effective biomarker for measuring the progress of a COVID-19 infection. Companies such as Beckman Coulter, Inc. and Roche Diagnostic have completed development work and received Emergency Use Authorization (EUA) from the FDA for in vitro diagnostic tests for IL-6. In these tests, IL-6 will serve as a biomarker for monitoring the severe inflammatory response in patients with a confirmed COVID-19 infection [46].

### 2.5. Glycans

Glycans, which are long chains of sugar molecules, are known to be one of the main components of the outermost surface of the virus. In the viral S protein, an examination of 66 glycosylation sites revealed that SARS-CoV-2 glycoproteins can contain up to 10 different glycans [47]. The structure of the glycans and the mechanism of glycan binding depends on the interaction between the viral pathogens and the hosts cells [48]. Glycans play a significant role in the binding of the virus to the host cell, and they can serve as biomarkers for recognizing a SARS-CoV-2 infection [49]. In a previous report about the swine zoonotic pandemic, it was found that porcine viral hemagglutinins can switch the binding sites in sialic acids [50]. This result demonstrates the significant role of glycans-binding during an infection. With an understanding of the unique structure of glycans found on SARS-CoV-2, glycans stand as a potential biomarker for the detection of the SARS-CoV-2 virus.

## 3. Factors that Affect the Development of Diagnostic Tests for SARS-CoV-2 

### 3.1. Materials Evaluation

A variety of samples, including those collected from the respiratory tracts, saliva, blood, and plasma, have been used for SARS-CoV-2 detection. Typically, non-invasive samples are collected from the upper respiratory tract (nasopharyngeal swabs (NP), oropharyngeal swabs (OP), anterior nasal swabs, mid-turbinate swabs, nasopharyngeal washes, and nasal aspirates) or the lower respiratory tract (sputum, BAL fluid, and tracheal aspirates) [18,19,51,52]. NP swabs and OP swabs are recommended for detecting the SARS-CoV-2 virus in the early stages of infection [53]. The average viral load for both swabbing techniques during the first 5 days of infection is in the range of 6.76 × 10^5^ copies per swab [54]. The viral load reaches a peak of 7.11 × 10^8^ copies per swab on day 5 [53,54]. No positive results were received from nucleic acid tests that were done using 27 urine samples and 31 serum samples [53]. In addition to molecular assays, rapid diagnostic tests, such as antigen testing methods, also use nasopharyngeal samples to detect the presence of SARS-CoV-2. Therefore, these swab samples are effective materials for detecting the SARS-CoV-2 virus during an active infection.

In contrast to the molecular approach, blood and plasma samples are typically used for serological tests. The viral concentration in the specimens gradually changes over time because of viral replication. The time dependency of the viral load concentration is a limitation associated with serological methods. In an experiment that tested 535 plasma samples from 173 infected patients, it was found that the concentration of antibodies increases shortly after an infection [36]. The median time for presenting the counts for total antibodies (Ab), IgG, and IgM was day 11, 12, and 14, respectively [36]. One week after symptom onset, the sensitivities of the antibody measurements were <40%. This percentage increased to 100% (Ab), 94.3% (IgM), and 79.8% (IgG) after 15 days [36]. Antigen tests use S and N proteins as the main viral antigen targets. A positive antigen test indicates an active infection. In the antibody detection approach, the IgG, IgM, and IgA antibodies related to SARS-CoV-2 are qualitatively detected. In addition, inflammatory biomarkers, such as interleukin-6 (IL-6), can be detected in COVID-19 patients. Therefore, blood and plasma samples can be used to indicate an active or previous infection in a patient.

### 3.2. Sensitivity, Specificity, and Accuracy

Currently, there is no universal testing kit for the detection of the SARS-CoV-2 virus, and different kits use different units of measurement. In many commercial testing kits and advanced laboratories, the success of a diagnostic test has been evaluated based on sensitivity, specificity, and accuracy. Sensitivity is defined as the probability that a positive result will be found when the SARS-CoV-2 virus is present in a sample. Specificity is defined as the probability of receiving a negative result when the SARS-CoV-2 virus is not present in a sample. Both sensitivity and specificity are calculated by the equations:

$$\mathrm{Sensitivity}\;=\;\frac{\mathrm{TP}}{\mathrm{TP}+\mathrm{FN}}\;\;\;\;\;\;\;\;\;\mathrm{Specificity}\;=\;\frac{\mathrm{TN}}{\mathrm{FP}+\mathrm{TN}}$$

where TP is true positive, FN is false negative, TN is true negative, and FP is false positive.

Some testing kits use the positive predictive value (PPV) and negative predictive value (NPV) to indicate the probability of a non-infected patient receiving a positive or negative test result, respectively [55].

## 4. Diagnostic Tests for Detection of SARS-CoV-2

A summary of the current techniques, sample materials, specific targets, and limits of detection (LOD) used by laboratories and biotechnology companies is presented in Table 1 and Table 2. There are two main approaches for detecting SARS-CoV-2. In the nucleic acid approach, the viral genome sequence is used to detect the SARS-CoV-2 virus. Different targets include the E gene, S gene, and Orf1ab gene. In the serological approach, both antigen and antibody tests are used. Antigen tests use S and N proteins as the main viral antigen targets. A positive antigen test indicates an active infection. In the antibody detection approach, the IgG, IgM, and IgA antibodies related to SARS-CoV-2 are qualitatively detected. This type of serological test can indicate a previous infection. In addition, inflammatory biomarkers, such as interleukin-6 (IL-6), can be detected in COVID-19 patients. Other emerging methods include imaging techniques and microfluidic devices.

### 4.1. Molecular Assays for Detection of Viral Nucleic Acids

Based on the available genetic sequence for SARS-CoV-2, viral ribonucleic acid (RNA) can be detected. Common methods for viral RNA detection include polymerase chain reaction (PCR)-based technologies, isothermal amplification techniques, CRISPR-based platforms, and high throughput sequencing technologies. These types of nucleic acid tests have become the most common methods used in CDC and clinical laboratories for the detection of SARS-CoV-2. The number of commercial companies that use different types of nucleic acid approaches for the detection of SARS-CoV-2 is shown in Table 1. The majority of samples used for nucleic acid testing are collected from the upper respiratory tract (nasopharyngeal swabs, oropharyngeal swabs, nasopharyngeal washes, and nasal aspirates) or the lower respiratory tract (sputum, BAL fluid, and tracheal aspirates).

#### 4.1.1. Polymerase Chain Reaction (PCR)

**Figure 2:**
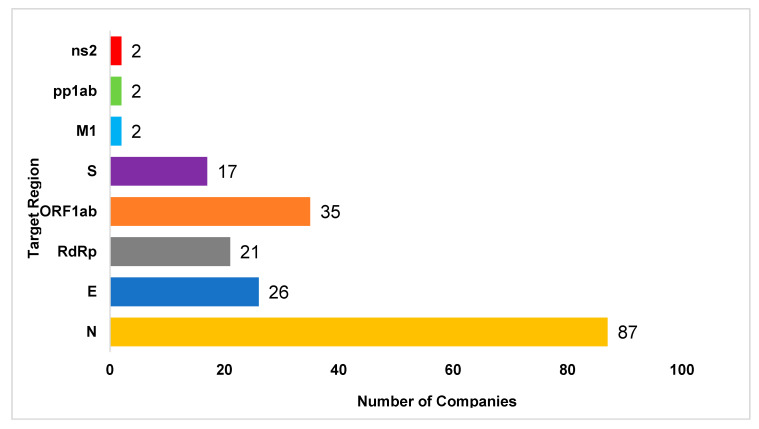
The number of commercial companies that use different target regions for designing the SARS-CoV-2 virus sequence in nucleic acid testing. The most common genes are the nucleocapsid gene, envelope gene, spike gene, ORF1ab gene, membrane gene, replicase polyprotein 1ab gene, and non-structural protein 2 gene. Among these, the most prevalent targeted gene is the nucleocapsid gene (73/163 companies). The scientific data was collected from the FDA website on 30 September 2020.

PCR-based technologies, which are molecular methods used for the detection of SARS-CoV-2, are able to amplify a few copies of small viral DNA/RNA segments into millions of copies [18]. The nucleic acid segments used in the tests come from specimen samples collected from the upper and lower respiratory tracts (nasopharyngeal swab, oropharyngeal swab, anterior nasal swab, mid-turbinate swab, nasal washes, nasal aspirates, and bronchoalveolar lavage) [18,19,51,52]. This technique is broken into two segments: (1) Designing the primer and probe alignment and (2) assay testing. During the design process, one primer is selected to detect a variety of coronaviruses including SARS-CoV-2. A second primer is then selected to detect only SARS-CoV-2. Different primers such as the ORF1a gene, ORF1b gene, ORF8, RdRp gene, N gene, and E gene are selected to design the gene sequence of SARS-CoV-2 [51,56]. The number of companies that use each specific target gene is shown in Figure 2. In the assay testing, there are three primary steps: denaturation, annealing, and extension. Among PCR-based technologies, RT-PCR is the most common method used by advanced laboratories and companies to detect SARS-CoV-2. In this approach, SARS-CoV-2 RNA is reverse transcribed into complimentary deoxyribonucleic acid (cDNA) strands through the exponential amplification of specific cDNA targets [52]. RT-PCR can be run according to a one-step or two-step procedure. The one-step method involves running the entire RT step in the same reaction tube as the PCR reaction [57]. The two-step method first requires the creation of a cDNA strand through the RT reaction. After the cDNA strand is formed, the PCR reaction is completed in a separate reaction tube, and the DNA concentration is measured using a fluorophore-quencher probe [18,19,51,52]. Although most laboratories use a singleplexed RT-PCR technique, the CDC has developed a multiplexed assay (CDC influenza SARS-CoV-2) that uses RT-PCR to detect the viral RNA of multiple viruses, such as SARS-CoV-2, influenza A, and influenza B, in a single test. In this assay, each primer and probe is selected to detect a specific gene sequence. The targeted sequences include the N gene for the SARS-CoV-2 virus, matrix (M1) gene for the influenza A virus, nsp2 gene for the influenza B virus, and RNaseP (RP) gene for human nucleic acid [58]. This assay can contribute to new developments in the detection of SARS-CoV-2 along with other infectious viruses.

Among nucleic acid tests, RT-PCR techniques have several advantages such as a low LOD, high sensitivity, and high specificity during the early stages of infection. Compared to other nucleic acid techniques, RT-PCR only requires samples that contain a small amount of the expressed gene. Specifically, the LOD in RT-PCR was estimated to be between 0.91–23 RNA copies/mL [59]. A second advantage of RT-PCR is its high sensitivity during the first few days of infection. During the first two weeks of infection, the sensitivity of RT-PCR was found to be higher than that of other techniques [60]. A third advantage of RT-PCR is its ability to be tailored to detect specific regions of the viral RNA. In a comparison between three different target regions (RdRp, E and N gene), Corman et al. claimed that RdRp regions in RT-PCR assays had the lowest LOD [51]. Therefore, the majority of RT-PCR tests use the RdRp region as the main target sequence. To further investigate the sensitivity of the RdRp region, Chan et al. compared the sensitivities of RdRp/Hel and RdRp/P2 assays [18]. When using different types of clinical specimens (i.e., respiratory tract specimens and non-respiratory tract specimens), they found that the sensitivities of the SARS-CoV-2 RdRp/Hel assays were significantly higher than the sensitivities of the RdRp-P2 assays. The total confirmed sensitivity of the SARS-CoV-2 RdRp/Hel assay was 43.6%, and the total sensitivity of the SARS-CoV-2 RdRp/P2 assay was only 28.2% [18]. The specificity of RT-PCR was 100% because of the unique genome sequences chosen in the design step [28].

Despite the benefits of RT-PCR, this technique also has some disadvantages. The timeline for detecting the SARS-CoV-2 virus through RT-PCR is limited by the period of infection. The number of virus particles present in a sample significantly decreases 3 weeks after infection occurs, and a low viral concentration limits the effectiveness of the test [60]. False negative results might occur depending on when a sample is collected or differences in patient immune systems. Another disadvantage is the prevalence of false negative tests in asymptomatic or recovered patients. Finally, PCR-based methods require long testing times (24 h or a few days), complex equipment, and well-trained technicians to administer tests and evaluate test results. These requirements limit the testing capabilities of hospitals and clinical laboratories using this diagnostic technique.

#### 4.1.2. Isothermal Amplification

- Reverse Transcription Loop-Mediated Isothermal Amplification (RT-LAMP)

**Figure 3:**
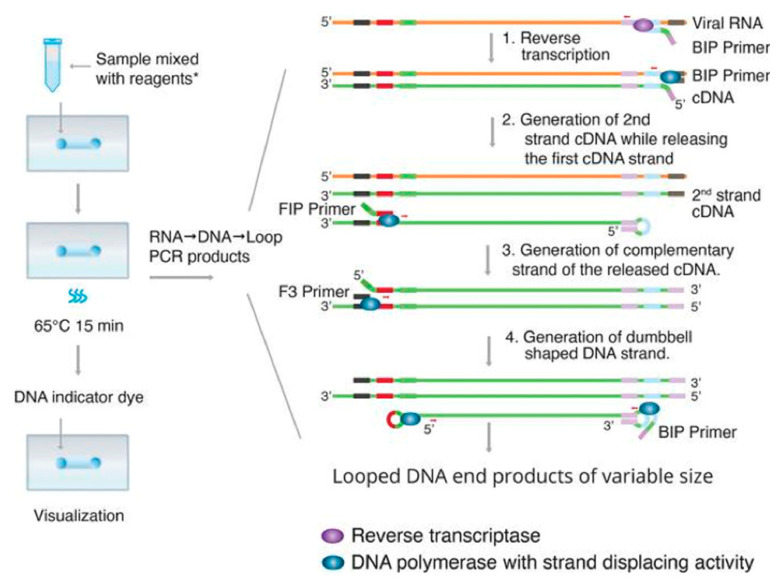
The main principle of the transcription loop mediated isothermal amplification (RT-LAMP) technique. RT-LAMP starts with the reverse transcription of the backward inner primmer (BIP). The BIP primer binds to the target sequence on the 3′ end of the RNA template and synthesizes a copy of the DNA strand (cDNA). Then, by using DNA polymerase, B3 primers bind to the side of the templates, generate the new cDNA strand, and release the first cDNA strand. This single strand of cDNA is then looped at the 3′ end and binds to itself. Next, the forward inner primmer (FIP) binds to the 5′ end of the strand and synthesizes a complementary strand by DNA polymerase. Then, the F3 primer binds to the end and generates a new double strand of DNA by DNA polymerase. The loop keeps running as a dumbbell-structure when the FIP or BIP primer initiates DNA synthesis again at the next target sequence location. The cycle can start at either the forward or backward side of the strand. When it starts, the strand undergoes self-primed DNA synthesis during the elongation stage of the amplification process. This amplification can be done in a short amount of time (approximately 1 h) and occurs at a constant temperature between 60–65 °C. Adapted by permission. Copyright of Linda C. et al. [66].

Another available nucleic acid testing method for detecting the SARS-CoV-2 virus is reverse transcription loop mediated isothermal amplification (RT-LAMP) [61,62]. The RT-LAMP technique amplifies the nucleic acid in one step using reverse transcriptase, DNA polymerase, and four to six oligonucleotides (oligo) of a certain architecture [63,64]. Specifically, some oligos containing a strand of DNA work as primers for reverse transcriptase. Other oligos are synthesized by DNA polymerase in the auto-cycling process, which is described in Figure 3. After the nucleic acid has been amplified, the new DNA products are repeatedly displaced and synthesized as a loop [63]. This auto-cycling process generates a variety of double-stranded looped DNA structures, which can be detected based on pH sensitivity [22], fluorescent response [65], and turbidity [62].

There are various advantages of the RT-LAMP approach in the detection of the SARS-CoV-2 virus. First, the detection time of SARS-CoV-2 by RT-LAMP is shorter compared to the traditional RT-PCR. According to Jiang et al., the process time to detect SARS-CoV-2 using RT-LAMP is 2 times faster than that for qRT-PCR [67]. Due to the formation of a double-stranded loop DNA structure, the denaturation steps required in RT-PCR are not necessary in RT-LAMP. Moreover, LAMP is a stable reaction where time-dependent inhibitors are tolerated. As a result, purification steps are not required [63,68]. Normally, the temperature of each step in RT-PCR is different, but the temperature of RT-LAMP is constant at 65 °C. Thus, the total reaction time for RT-LAMP is significantly shortened. Second, despite the removal of the RNA isolation step, the specificity of RT-LAMP is high compared to RT-PCR. In an experiment by Dao et al., the specificity of RT-LAMP was 99.5%. The sensitivity was only 86%, however, when the cycle threshold (CT), which is the cycle number when the fluorescence of a PCR product can be detected above the background signal, was less than 30 [69]. Third, the RT-LAMP technique does not require complicated equipment such as a thermal cycler with real time fluorescence measurement capabilities. After incubation at 65 °C, positive results from patient samples can be determined in 30 min by observing a color change from red to yellow [64]. Instead of using a specific instrument to identify the color change, cameras in mobile phones, copy machines, and plate scanners with spectrophotometric quantification capabilities can be utilized. Finally, the materials used for RT-LAMP do not need to be refrigerated and can be stored at room temperature [67]. 

Despite many benefits, LAMP techniques have several limitations. The limitations are mainly associated with the low sensitivity of single tests and the optimization of primer design and reaction conditions. For a RT-LAMP test with RNA extraction, Rodel et al. found that the sensitivity of RT-LAMP was 75% despite there being no false positive results. The sensitivity was 86.4% when RT-PCR was used to identify the E gene [70]. When the RT-LAMP and RT-PCR techniques were used together, the sensitivity and specificity increased to 100% [69,70]. To improve sensitivity, the RT-LAMP assay could be used with a specific RT-qPCR Ct number. The second disadvantage of RT-LAMP is that the process for designing primers and probes capable of targeting specific gene sequences can be complex [63]. As a result, primer optimization might be limited. Third, RT-LAMP has only been able to detect SARS-CoV-2 in patients with a high viral load. A low RNA input might lead to false negative results. In one study by Jiang et al. on primer design for LAMP techniques, the virus could not be detected in four positive patients when the volume of RNA input was 2 µL. This amount was 2.5 times less than the amount of RNA used for qRT-PCR (5 µL) [67].

- Transcription—Mediated Amplification (TMA)

Similar to the principle of RT-LAMP, transcription-mediated amplification (TMA) is an isothermal amplification technology that uses reverse transcriptase RNA polymerase, RNaseH, and a T7 promoter-labelled target—a specific primer to detect nucleic acids [71,72]. One of the major differences between TMA and other nucleic acid techniques (PCR) is the Ribonuclease H (RNaseH) activity in the reverse transcriptase reaction [71]. RNaseH is an enzyme that can remove RNA from a cDNA strand. The RNaseH activity in TMA performs the function of the heat denaturing step in RT-PCR [73]. For TMA, the enzyme and primers are first combined together to produce a hybrid fragment of RNA:DNA with a T7 promoter. This hybrid is considered the first primer. The target RNA strand from the hybrid fragment is then degraded by RNaseH. Subsequently, the RNA strand is reverse transcribed into a single-stranded cDNA molecule with a T7 promoter. Finally, a double stranded DNA molecule is generated by the transcription of T7 RNA polymerase into RNA amplicons. This process is repeated to generate an exponential amount of RNA amplicons in less than 1 h [66]. The conjugation between single-stranded nucleic acid torches and RNA amplicons can be detected in real time by a fluorescence emission [66]. At Hologic’s Panther Fusion company, it was found that TMA tests for SARS-CoV-2 had a better analytical sensitivity compared to RT-PCR based tests. In their results, the LOD for SARS-CoV-2 was 5.5 × 10^4^ copies/mL, and the sensitivity was 98.1% in TMA and 96.2% in PCR [74]. RT-PCR tests done by the CDC were unable to detect the SARS-CoV-2 virus at this viral concentration [74]. Moreover, due to the ability of TMA to amplify a specific target region of RNA or DNA, this method has been used to detect other pathogens such as the Hepatitis C virus [75] and Hepatitis B virus [76].

#### 4.1.3. CRISPR-Based Nucleic Acid Detection

**Figure 4:**
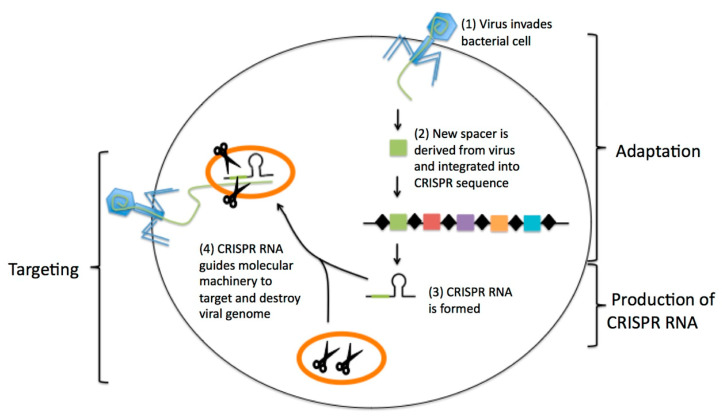
The principle of the clustered regularly interspaced short palindromic repeats (CRISPR)-based technique for the detection of SARS-CoV-2. CRISPR/Cas12 and CRISPR/Cas13 are used to detect the viral RNA of SARS-CoV-2. The Cas13 complex binds to the target sequences. This binding activates the general nuclease enzyme activity of Cas13 to cleave the target sequence. The RNA is then detected by a fluorescence signal. The activity of Cas12 is similar to the activity of Cas13. One difference between the two is the position of the nuclease enzyme. Adapted by permission. Copyright of Barrangou R. et al. [83].

Other molecular techniques that are used to detect SARS-CoV-2 include clustered regularly interspaced short palindromic repeats (CRISPR) and CRISPR-based (Cas) nuclease systems. A CRISPR is a segment of DNA with repetitive base sequences that are involved in the prokaryotic adaptive immune system [77]. The specific process of CRISPR-associated enzymes is described in Figure 4. Based on this system, a CRISPR/Cas tool has been engineered to edit the genome in mammalian cells. Currently, there are three types of Cas nucleases: Cas3, Cas12, and Cas13. CRISPR-Cas3 employs the endonuclease Cas3 enzyme and a multiprotein complex for antiviral defense [78,79]. Cas3, an intermediate enzyme, is recruited to targeted DNA and mediates DNA cleavage [78]. Once the recruitment of Cas3 is activated, the target DNA is degraded by 3′ to 5′ exonuclease activity [79,80]. During the process of detecting the SARS-CoV-2 virus, the Cas3 enzyme contributes to the cleavage of targeted DNA. CRISPR-Cas12 and CRISPR-Cas13, which are Class II CRISPR-Cas systems, consist of a single nuclease that is related to the cleavage of nucleic acid targets [78]. Cas12 is defined as an RNA-guided DNase that can degrade single-stranded DNA (ssDNA) and induce ssDNA cleavage. This mechanism activates a fluorescence emission in the cleavage [25]. Cas13 is defined as a non-specific RNase that does not activate until the RNA targets are programmed to bind together. Both CRISPR-associated enzymes can be utilized for targeting and cutting sequences of RNA. For instance, the DNA endonuclease targeted CRISPR trans reporter (DETECTR) technique utilizes a CRISPR-Cas12 assay and the RT-LAMP technique for RNA extraction [81]. Moreover, the specific high-sensitivity enzymatic reporter un-LOCKing (SHERLOCK) technique, utilizes a CRISPR-Cas13 assay with isothermal nucleic acid amplification methods [82]. Therefore, the combination of Cas nucleases with other nucleic acid tests can speed up the development of diagnostic tests for COVID-19.

CRISPR-based technologies have several advantages for the detection of SARS-CoV-2. First, DETECTR based tests have a shorter process time compared to RT-PCR based tests. In a study completed by Broughton et al., the detection time for a DETECTR assay was 45 min, while the time required for RT-PCR was 4 h [81]. Second, the LOD of the DETECTR technique is low and the sensitivity, specificity, and accuracy of the tests are high. The LOD of the DETECTR approach is 10 copies RNA/µL, and the LOD of RT-PCR tests is 0.91–23 copies RNA/µL [25,81]. Moreover, the precision of the measurements used in CRISPR-based platforms was predicted as 95% for positive tests and 100% for negative tests [81]. Compared to nucleic acid tests, such as RT-PCR and mNGS, CRISPR-based assays have higher specificity and sensitivity. According to a study by Hou et al., CRISPR-based assays could obtain a sensitivity of 100% with no false positive cases. PCR based tests only detected 90.4% of positive cases for a CT range between 28.8 and 40.4 [84]. Third, the editing genome function of CRISPR technologies allows this platform to be used with other nucleic acid approaches to improve the detected signals. For instance, the association of the Cas13 system and isothermal amplification, which is called the SHERLOCK technique, is performed using two separate steps (1) isothermal RPA and (2) T7 transcription and Cas13-mediated collateral cleavage of a single-stranded RNA reporter [85]. A part of RNA, which is not bound to the target, will activate a fluorescence signal [78]. With the initiation of complex binding and the cleavage of nucleic acid targets during genome editing, the detected signal in the SHERLOCK technique can be improved. Fourth, CRISPR-based technologies that contain a lateral flow strip produce test results that can be read with the naked eye. Currently, the combination of Cas3/Cas12/Cas13 with a nucleic acid platform (LAMP) in a lateral flow assay is being used to develop a rapid and sensitive diagnostic test for the SARS-CoV-2 virus [81,86].

The use of CRISPR-based technologies also comes with several challenges. First, there are currently no portable CRISPR-based devices for detecting SARS-CoV-2. A previous study has shown that a portable SHERLOCK device was able to detect the single stranded RNA of the Dengue and Zika viruses [87]. Although this technology has not been adapted for the SARS-CoV-2 virus, the success of CRISPR methods in detecting other pathogens shows that this technology has the potential to be used with SARS-CoV-2. Second, the off-target effects of CRISPR platforms, which result from unintended point mutations, deletions, insertions, inversions, and translocations in the assay, might reduce the accuracy of detecting SARS-CoV-2 nucleic acids. In a study by Anzalone et al., the prime editing method associated with the Cas9 protein and reverse transcriptase can describe the ingenious path for a strand of DNA and improve the efficiency of CRISPR-based methods. Even though this editing approach has not been developed for the SARS-CoV-2 virus, it has the potential to improve the editing process of DNA templates [88,89]. Other disadvantages are related to mismatch between RNA and target nucleotides [88].

#### 4.1.4. High-Throughput Technologies

High-throughput sequencing technologies, such as next generation sequencing (NGS) and amplicon/metagenomic based sequencing (MinION), are able to sequence several DNA molecules at one time [90]. This technique generates large datasets and gives insight into the cellular genomic and transcriptomic characteristics of various diseases. These techniques are also used to develop diagnostic tests for the SARS-CoV-2 virus.

- Next Generation Sequencing (NGS)

NGS is a platform that is used along with other techniques to develop the sequence of several small DNA fragments simultaneously [91]. Specifically, when NGS is used with bioinformatic techniques, it has been successful in recognizing and analyzing pathogens. With this technique, one can extract information from a genome sequence while simultaneously identifying the presence of a virus in a sample [91]. This commercial technique was developed by Illumina [92]. It can detect the presence of multiple strains of coronaviruses in a sample and determine the presence of different pathogenic organisms. Specifically, NGS is able to enrich large amounts of oligo probe panels by hybrid capture with a comprehensive profile of target regions. In these oligo probes, highly mutagenic regions can be used to determine how viruses, especially RNA viruses, have evolved [92]. According to Illumina databases, this technique has a sensitivity 98% and an LOD of approximately 1000 copies/mL [93]. Some of the main disadvantages of NGS include the difficulty of selecting a genome sequence platform, the need for a highly-trained technician, and the use of computational bioinformatics that requires the collection of large datasets.

- Amplicon-Based Metagenomic Sequencing

Another high-throughput sequencing technique that is used to detect SARS-CoV-2 is called amplicon-based metagenomic sequencing. Similar to NGS, this technique uses a combination of the base amplicon and metagenomic sequencing (MinION) to detect and analyze the microbiome in nasopharyngeal swabs [94]. This amplicon-based sequencing technique contributes to the detection of the SARS-CoV-2 virus by improving contact tracing, molecular epidemiological studies, and studies on viral evolution. This strategy can be used to determine the divergence of a gene sequence. Metagenomics approaches, including sequence-independent single primer amplification (SISPA), are able to provide additional confirmation on the divergence of gene sequence. Compared with other amplification techniques, the combination of amplicon-based sequencing and SISPA techniques would provide a lower rate of mutation and recombination with other human viruses. Mutation and recombination can have significant effects on vaccine and antiviral efficacy. This method was also used in the West African Ebola virus outbreak to address the characteristics of the viral infection. Although amplicon-based metagenomic sequencing has the potential to be used for the detection of SARS-CoV-2, there are several limitations associated with this method. While this method has high accuracy, sensitivity, and specificity, the time required for viral detection is long (8 h) compared to some of the other nucleic acid approaches.

### 4.2. Rapid Diagnostic Tests (RDT)

Due to the limitations of molecular methods, new strategies that use antigens or antibodies in patient samples to detect SARS-CoV-2 were developed.

#### 4.2.1. Antibody Testing

The typical sample materials used for antibody testing are blood from finger pricks and plasma. To detect the presence of the SARS-CoV-2 virus, the samples are tested to detect patient antibodies (IgG, IgM, and IgA) that are specific to viral antigens. The targets used for different serological methods are presented in Table 2. IgG, IgM, and IgA antibodies are the main targets used for antibody testing. The viral concentration in the specimens gradually changes over time because of viral replication. Therefore, the concentration of the viral load is a limitation associated with serological methods. Currently, there are various point-of-care (POC) diagnostic platforms that are used for SARS-CoV-2 detection. These platforms include rapid lateral flow diagnostic tests, enzyme-linked immunosorbent assays (ELISA), and proteome peptide microarrays (PPM).

- Enzyme-Linked Immunosorbent Assay (ELISA)

ELISA is commonly used for the detection and quantification of proteins, antigens, antibodies, glycoproteins, and hormones [95,96]. There are three different types of ELISA assays that are used to detect SARS-CoV-2. These assays include direct ELISA, indirect ELISA, and sandwich ELISA. Both direct and indirect ELISA use antigens/antibodies to detect the SARS-CoV-2 virus. In sandwich ELISA, the viral antigens are detected when they bind to two different antibodies (capture antibodies and detection antibodies). All three techniques start by coating 96-well plates overnight with human SARS-CoV-2 proteins from human samples. These proteins bind to the polystyrene plate by an enzyme-substrate reaction [97]. When the viral protein is present, antiviral antibodies from the patient samples bind to the plate. The antibody-protein complexes can be detected with an additional tracer antibody that produces a color signal [96]. The average time to receive a result is approximately 2 to 5 h. In addition to IgG, IgM, and IgA, plasma cytokines, such as IL-6 antibodies, are also used to detect the presence of SARS-CoV-2 using ELISA [98].

Compared to the nucleic acid testing methods, the ELISA technique has several advantages for detecting SARS-CoV-2. These advantages include high sensitivity and specificity when an infection has occurred over 2 weeks prior to testing. In a recent study, Xiang et al. compared the sensitivity, specificity, and accuracy of SARS-CoV-2 tests that were developed to detect the presence of human IgG and IgM antibodies [99]. The sensitivity of tests using IgG antibodies and the sensitivity of tests using both IgG and IgM antibodies were 82.5% and 87.3%, respectively. The specificities of all three assays (only IgG, only IgM, and a combination of IgG and IgM) were 100%. The accuracy of the tests increased to 91.8% when both antibody types, IgG and IgM, were used [99]. Zhang et al. also showed that the sensitivities of ELISA tests using IgG and IgM were high 2 weeks after infection. On day 0 of sampling, the concentrations of IgG and IgM were too low, and the presence of the SARS-CoV-2 virus could not be detected. On day 5, the percentage of positive results from IgG tests increased from 50% to 81%. The percentage of positive results from IgM tests increased from 81% to 100% [100]. A second advantage of ELISA is a shorter processing time compared to nucleic acid testing methods. Third, the use of gold nanoparticles (AuNP) with ELISA improves the sensitivity for colorimetric detection of an antigen. Ciaurriz et al. found that the sensitivity of AuNP-based ELISA was 5 times greater than the sensitivity of standard plate type ELISA. The limit of detection, however, was 3 times lower for AuNP-based ELISA compared to standard plate type ELISA [101]. In addition, results obtained using AuNP-based ELISA are easier to interpret and can be detected by the naked eye.

A comparison between the different types of ELISA-based assays shows that each type has distinct advantages for detecting the presence of the SARS-CoV-2 virus in infected patients. Since direct ELISA only uses a primary antibody, it reduces the risk of cross-reactivity with a secondary antibody. The procedure for direct ELISA is also faster because there are fewer steps. Indirect ELISA is advantageous because it is very flexible and different primary antibodies can be selected. Sandwich ELISA has the highest sensitivity among the three types. The use of two monoclonal antibodies improves the sensitivity and specificity of the test for the SARS-CoV-2 nucleocapsid protein. In a previous study by Fung et al., the combination of monoclonal antibodies (mAbs) in a sandwich ELISA assay enabled differentiation and correct identification of the MERS-CoV S protein and N protein [102].

In addition to its advantages, there are also several limitations of using ELISA for the detection of SARS-CoV-2. First, some commercial testing kits are only intended for research applications and have not been used in clinical settings yet. Second, monoclonal antibodies cannot identify the mutated virus in ELISA. According to Niikura et al., one monoclonal antibody can only identify three of the four subtypes of the Ebola virus. As a result, it is possible that variations in the SARS-CoV-2 virus may lead to cross-reactivity [103]. The SARS-CoV-2 virus structure has six different subtypes, so a false positive or negative result could possibly occur.

- Chemiluminescence Immunoassay (CLIA)

Chemiluminescence immunoassay is another technique used to determine the presence of antibodies in patient serum. Currently, this technique uses magnetic, protein-coated microparticles. The principle of CLIA is similar to the principle of direct ELISA. The main difference is the use of luminescence to read the results. There are several advantages of the CLIA method. First, due to the high signal intensity and absence of interfering emissions, CLIA can obtain higher sensitivities and specificities compared to other serological methods including LFIA and ELISA. For the measurement of IgG/IgM, the pooled sensitivity of CLIA was 97.8%. The sensitivity of ELISA was only 84.3%, and the sensitivity of LFIA was only 66% [104]. The use of magnetic and protein-coated microparticles with CLIA increases the sensitivity of the technique. According to the Liu et al., when using a microparticle CLIA approach, the sensitivity of the IgM measurement was 72.3%, and the sensitivity of the total antibody measurement was 90.8% [105]. However, when using a peptide-based magnetic CLIA assay, the sensitivity of the IgG and IgM measurements are only 71.4% and 57.2%, respectively [106]. Some of the significant limitations of CLIA include the high cost, complex instrumentation, and highly trained technicians.

- Lateral Flow Immunoassay (LFIA)

**Figure 5:**
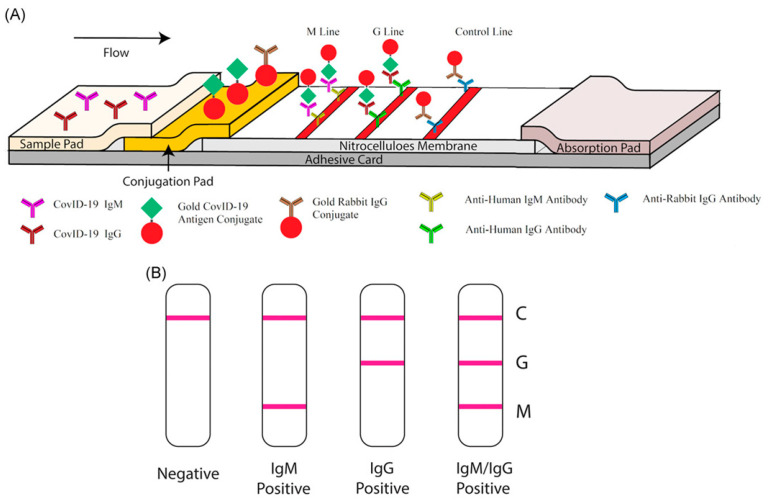
Lateral flow immunoassay for the detection of anti-SARS-CoV-2 antibodies [21]. The sample fluid flows laterally along the strip, which includes different zones such as conjugation pad, adhesive pad, and absorption pad. The conjugation pad includes antibodies for the target analyte and antibodies conjugated with signal molecules (fluorescent particles and gold particles). The nitrocellulose membrane contains the testing lines (IgG and IgM lines) and the control line. The last zone is the absorption pad, which prevents the backflow of the liquid. Adapted by permission. Copyright of Li Z. et al. [21].

Lateral flow immunoassay (LFIA) is a qualitative chromatographic assay that determines whether a target analyte, such as a biomarker, is present in an unknown sample. The typical sample materials used with this technique are blood from finger pricks, saliva, urine, and nasal swab fluids. To detect the presence of the SARS-CoV-2 virus, these samples are tested to detect patient antibodies (IgG, IgM, and IgA) that are specific to viral antigens in various targeted regions. The target antigens include S glycoproteins (S1 and S2 subunits, RBD) and N proteins [64,107]. LFIA is typically constructed as a strip including a sample pad, a conjugate pad, a membrane, a detection zone, and an adsorbent pad (Figure 5). The principle of LFIA is based on the movement of a fluid sample through different zones of a strip via capillary forces. To detect SARS-CoV-2 related antibodies, a viral antigen is conjugated to gold or fluorescent particles. The antibody in the patient sample binds to the viral antigen, which has been immobilized on the conjugate pad. If SARS-CoV-2 antibodies are present in a sample, they will bind to the gold-conjugated SARS-CoV-2 antigen. The antibody-antigen complex then flows to the capture zone of the device. In the capture zone, the antibody-antigen complex binds to another antibody which induces color formation. Many advanced laboratories and companies are developing kits based on the LFIA approach. These kits use different containers, such as cassettes and casings similar to those used for pregnancy tests.

There are several advantages of using LFIA to determine the presence of the SARS-CoV-2 virus through antigen or antibody testing. First, LFIA is able to produce results quickly. Specifically, LFIA takes approximately 10 to 30 min to detect SARS-CoV-2. The detection time for traditional ELISA is 2 to 5 h. Second, the sensitivity and accuracy of LFIA for the detection of SARS-CoV-2 increases 2 weeks after infection. According to Adam el al., the sensitivity of a LFIA device increased from 61% to 88%, 10 days after the onset of symptoms [23]. In another study by Niikura et al., the sensitivity of LFIA was found to be 100%, 3 weeks after the onset of symptoms [107]. Third, LFIA is inexpensive, and results can be detected by the naked eye.

Despite these benefits, there are some concerns with the use of LFIA in commercial products [108]. First, the sensitivity of LFIA was low compared to other serological methods. When using the same amount of sample material, LFIA had a sensitivity of 66%. This sensitivity was lower than other serological methods, which had sensitivities greater than 80% [104]. A comparison between different types of LFIA kits showed that the sensitivities of commercial LFIA kits (average 65%) was lower than the sensitivities of kits used in laboratories (average 88.2%) [104]. Second, it can be challenging to control the velocity of the fluid and the capillary forces during the process of antigen capture. Third, a previous report described that the pore size of the porous media can limit the fluid flow [109]. Fourth, the analysis time depends on the nature of the sample. For instance, storage conditions, fluid viscosity, and surface tension can all impact the sample analysis time [110]. Prolonged storage can possibly lead to false negative results. In a study by Wu et al., nine serum samples, which were collected from two patients with SARS-CoV-2, were stored for more than 9 days at −20 °C before an antibody response test was run. The results showed that two of the nine samples, which were taken on day 2 and 7 after symptom onset, showed no antibody response. The rest of the samples all successfully showed the correct results.

#### 4.2.2. Antigen Testing

Antigen testing is another approach used to detect the presence of the SARS-CoV-2 virus during an active infection. An evaluation of the most recent authorized SARS-CoV-2 antigen tests that were issued an EUA by the FDA are presented in Table 3. According to recent literature reports and commercially available product manuals, N proteins and S proteins were the most common targets used in antigen-based testing and serological methods. According to Baker et al., glycans are used as the capture unit when detecting SARS-CoV-2 S proteins with the LFIA approach [50]. Similar to antibody testing, antigen testing is performed using LFIA in two main forms (singleplex and multiplex). The typical sample materials used are nasal swabs and nasopharyngeal swabs. To determine the presence of the SARS-CoV-2 virus, these samples are tested to detect specific antigens in various targeted regions (S glycoproteins and N proteins). Currently, the EUA and FDA have approved N proteins as a target region for antigen testing kits. The LOD for N proteins is within the range of 1.0 × 10^2^ TCID_50_/mL to 4.5 × 10^5^ TCID_50_/mL. The advantages of antigen testing include rapid results, high sensitivity and specificity, inexpensive cost, and naked-eye result readout. According to a report of approved antigen testing kits from the FDA, the total time needed to receive results is less than 20 min. The sensitivity of these testing kits ranges from 84% to 97.6%, and the specificity is 100% based on a report from CDC [46].

#### 4.2.3. Proteome Peptide Microarray (PPM)

The proteome microarray technique is typically used to investigate the interactions of antibodies and proteins at the amino acid level [26,111]. This technique provides information on viral protein epitope identification and specificity mapping for the binding of antibodies to SARS-CoV-2 proteins. Recently, high-density peptide microarrays have been used to analyze the interaction profiles of antibodies in the human serum [26]. By providing an understanding of antibody responses to SARS-CoV-2 proteins, this technique can help determine the infection signals. Several commercially available antibodies that were used for SARS-CoV are also being used for SARS-CoV-2. The main advantage of the PPM method is that it can provide a holistic view of antibody responses to viral proteins. According to Jiang et al., the PPM method can identify the responses of IgG/IgM antibodies to N, S1, and S2 proteins on the SARS-CoV-2 virus. As a result, 100% of patients had IgG/IgM responses to the virus at the N protein and S1 protein targets. Meanwhile, the S2 target did not receive any responses [112]. Despite some advantages, there are several limitations with this technology. First, the epitopes of several SARS-CoV-2 antibodies may have been modified by post-translational changes or conformations. These modifications may affect the accuracy of the detection [26]. Second, many new strains of SARS-CoV-2 have been identified [113]. The test results may change depending on which viral strain is present in the sample. Finally, this technology has only been used for research, and it has not been approved for clinical diagnostic tests.

### 4.3. Medical Imaging

Computed tomography (CT) scans can also be used in the clinical diagnosis of SARS-CoV-2. This technique is used because it has a higher sensitivity than the currently available RT-PCR methods [19,114,115,116]. The detection of the virus in a patient is based on cross sectional X-ray images of the chest. New images are captured during each stage of infection (early stage, intermediate stage, and late stage) [116]. In CT scans, bilateral and peripheral ground-class opacities were found to be common symptoms of the viral infection. The changes in the lung images are analyzed in light of how much time has passed since symptoms first appeared. For patients who experienced acute-phase diffuse alveolar damage (≤7 days from the onset of respiratory failure), the SARS-CoV-2 virus was found in the pulmonary pneumocytes, ciliated airway cells, and upper airway epithelium. The maximum change in lung shape and the highest frequency of ground-glass opacities were observed 10 days after the onset of symptoms [116]. According to Yang et al., patient chest exams via X-ray demonstrated the highest positivity rate (100%) 8 to 14 days after the onset of symptoms [117]. This technique is not a viable option if a patient has pre-existing lung complications. In addition, this method is not suitable for asymptomatic carriers. Finally, it requires highly trained personnel and sophisticated instrumentation to be performed and evaluated.

### 4.4. Potential Diagnostic Tests

#### 4.4.1. Vertical Flow Assays (VFA)

Another approach that can be used to detect SARS-CoV-2 is a vertical flow assay (VFA). This technique is similar to a lateral flow assay, but it uses a vertical flow pattern instead. Both assays require the formation of an antibody-antigen complex, immobilization of the capture antibody onto a readout layer, and the immobilization of the labelled detection antibody for the production of a color signal [109]. The main advantages of vertical flow assays include the use of external forces, such as gravitational forces and capillary forces, and the ability to easily multiplex the assay. Additionally, the VFA sensor response is faster than that of a lateral flow assay, and the test results can be evaluated by untrained users [108]. The pore size of the membrane and the optimized flow rate determines the sensitivity of the test. VFA has a faster detection time than LFA because of the immediate reaction between the specific antigen and the conjugated detection antibody. The signal can be detected with the naked eye, or it can be quantified using imaging techniques, such as those used in smartphone readers [108]. According to Chen et al., vertical flow can increase the flow rate and decrease the required pore size of a membrane. As a result, vertical flow methods can be up to 5 times more sensitive than lateral flow methods [109]. Vertical flow is an alternative configuration that can address the pore size, velocity control, and time limitations associated with lateral flow. Therefore, VFA can possibly be used to detect the SARS-CoV-2 virus.

#### 4.4.2. Microfluidic Devices

Microfluidic devices can also be considered for the diagnosis of SARS-CoV-2. These devices typically consist of a centimeter-scale chip with micrometer-scale channels and chambers [28]. Microfluidic devices have been constructed using materials such as paper, polymers, and glass. The advantages of microfluidic devices include the ability to provide quick results, portability, and the ability to use small sample volumes. Previous research has shown that microfluidic platforms are able to detect pathogens such as the human immunodeficiency virus (HIV), the hepatitis B virus (HVB), and the Zika virus (ZIKV) [118]. Moreover, these devices have been developed in combination with methods such as RT-PCR, RT-LAMP, LFIA, and ELISA. In a study completed by Qui et al., the combination of RT-PCR and microfluidics was able to detect the H1N1 virus in less than 30 min. Standard RT-PCR techniques require up to 24 h to detect the H1N1 virus. In this work, the capillary tubes in the microfluidic devices were fabricated using injection molding. A smartphone was then used to image and quantify the fluorescent signal from the diagnostic test [119]. Microfluidics-based approaches have a high potential to be used for the detection of SARS-CoV-2.

## 5. Test Considerations

Although disadvantages and advantages are described for each approach, no universal diagnostic test is available to detect the SARS-CoV-2 virus. There are still several factors that need to be considered before a universal test can be developed. These factors include the analytical results, the conditions for sample storage, and the population samples. First, the analytical results such as the sensitivity, specificity, and accuracy of different testing methods depends on how much time has passed since the infection occurred. Nucleic acid testing can identify the viral load during the early stages of infection, serological testing is able to diagnose patients who were previously infected or are actively infected, and the imaging techniques have been developed for patients with mild to severe symptoms [120,121]. Second, there is a lack of information regarding the external storage conditions for human samples. Storage conditions, such as temperature and storage time, can affect the precision of diagnostic tests [122]. Different sample types require different collection methods and storage conditions [123]. In a previous study by Wu et al., two false negative results for SARS-CoV-2 occurred in a LFIA-based test with long sample storage times [107]. The incorrect results might have originated from the degradation of proteins while the sample was in storage. According to a study by Shenouda et al., the denaturation process of nucleotides, carbohydrates, small peptides, and free amino acids in proteins are significantly impacted by cold temperatures [124]. Information about the external storage conditions for samples should be studied to increase the accuracy of test results. In another study by Bevins et al., it was found that antibody proteins can be kept in storage at freezing temperatures with desiccants [125]. More information about sample collection and storage should be indicated to increase the accuracy of the result. Third, a standard sample size for each technique has not been determined due to the lack of a large population to study and a lack of information on asymptomatic cases. In a comparison between blood and swab samples in RT-PCR, the positive rates were 15%–30% and 14%–38%, respectively [126]. However, the samples were obtained from only 23 patients for blood tests and 15 patients for swab tests [126]. Samples should be collected from a wider population to validate this result. Third, there are still many questions surrounding viral replication and evolution as a result of re-infection cases.

## 6. Conclusions

Over the last few months, industrial companies and scientific laboratories have developed a variety of different tests to diagnose patients with the SARS-CoV-2 virus. The latest research is focused on developing efficient tests that are inexpensive, rapid, reliable, and have high sensitivity, specificity, and accuracy. Most of the published papers and commercial products have explored different types of molecular assays, serological methods, and imaging techniques for detecting SARS-CoV-2. RT-PCR is the most common nucleic acid technique for detecting viral RNA for SARS-CoV-2. Different types of amplification assays, including CRISPR-related technologies and other high-throughput sequencing methods, have been developed to increase the sensitivity and specificity of these diagnostic tests. Moreover, POC testing kits that are rapid and simple-to-use have also been developed.

Despite minor drawbacks, some of the diagnostic techniques work well in clinical settings. Other newly developed techniques demonstrate promise for clinical use. By using the knowledge gained from previous outbreaks and the currently available strategies, diagnostic tests for SARS-CoV-2 will continue to be developed and improved.

## Figures and Tables

**Table 1:** Available laboratory-based molecular, serological, and immunological diagnostic test for the severe acute respiratory syndrome coronavirus-2 (SARS-CoV-2).

Techniques	Testing Materials	Platform	The Amount of Sample/Sample Size	Detection Workflow	Detection Target	Limit of Detection (LOD)
RT-PCR [18]	Sputum, nose, and throat swabs with and without viral transport medium	Nucleic acid			RdRp genes	3.6 copies per reaction
					E genes	3.9 copies per reaction
					N genes	8.3 copies per reaction
					RdRp-P2	21.3 copies per reaction
					RdRp-Hel	11.2 copies per reaction
Real time RT-PCR [19]	Tear and conjunctival secretion	Nucleic acid	21 patients with common-type and nine patients in severe symptoms		Viral RNA in conjunctivitis	N/A
RT-LAMP [20]	Viral RNA	Nucleic acid	N/A	N/A	Primer probe	LOD in the optimization: 100 copies/reaction in triplicate
Lateral flow immunoassay [21]	Human blood	IgG/IgM Antibody	525 cases including 397 positive tests confirmed with PCR and 128 non-infected tests	Around 15 min	Spike protein	N/A
Isothermal LAMP-based [22]	Respiratory samples	Nucleic acid			ORF1ab gene	17 copies/µL
ELISA [23]	Plasma samples from healthy blood donors, organ donors, plasma samples from upper respiratory tract swab	IgG/IgM antibody	90 samples including 50 negative and 40 positive	90 min for observance 5–6 h	Spike protein	
LFIA [23]		IgG/IgM antibody	39–165 individual plasma samples			
Sandwich ELISA [24]	Pulmonary sarcoidosis after ablation	IgG/IgM antibody			Nucleocapsid protein	100 ng/mL
CRISPR-Cas12 [25]	Saliva samples	Nucleic acid			RNA fragments with RdRp, ORF1b, and ORF1ab genes	ORF1ab: 10 copies/µL
Proteome Microarray [26]	Serum samples	Antibody			Antibodies	94 pg/mL

**Table 2:** The general list of cross-reactivity result that have been tested from diagnostic tests.

Technique	Platform	Target	Sensitivity	Specificity	Accuracy	
					PPV	NPV
Alinity I SARS-CoV-2 IgG (Abbott)	High-throughput CMIA	Nucleocapsid	100%	99.9%	84%	100%
Architect SARS-CoV-2 IgG (Abbott)	High-throughput CMIA	Nucleocapsid	100%	99.6%	92.9%	100%
Anti-SARS-CoV-2 Rapid Test (Autobio)	Lateral flow	Spike	IgG: 99.0% IgM: 95.7% Combined: 99.0%	IgG: 99.4% IgM: 99.7% Combined: 99%	84.4%	99.9%
Platelia SARS-CoV-2 Total Ab (Bio-Rad Laboratories, Inc.)	ELISA	Nucleocapsid	92.2%	99.6%	91.7%	99.6%
qSARS-CoV-2 IgG/IgM Rapid Test (Cellex, Inc.)	Lateral Flow	Spike and Nucleocapsid	93.8%	96.0%	55.2%	99.7%
DPP COVID-19 IgM/IgG System (Chembio Diagnostic Systems, Inc.)	Lateral Flow with Reader	Nucleocapsid	IgM: 77.4% IgG: 87.1% Combined: 93.5%	Combined: 94.4%	46.8%	99.6%
LIAISON SARS-CoV-2 S1/S2 IgG (DiaSorin)	High-throughput CMIA	Spike	97.6%	99.3%	88%	99.9%
SARS-CoV-2 ELISA IgG (EUROIMMUN)	ELISA	Spike	90.0%	100%	100%	99.5%
COVID-19 ELISA Antibody Test (Mount Sinai Hospital Clinical Laboratory)	2-Step ELISA	Spike	Combined: 92.5%	100%	100%	99.6%
VITROS Anti-SARS-CoV-2 IgG test (Ortho-Clinical Diagnostics, Inc.)	High-Throughput CLIA	Spike	IgG: 90.0%	IgG: 100%	100%	99.5%
VITROS Immunodiagnostic Products Anti-SARS-CoV-2 Total Reagent Pack and Calibrator (Ortho-Clinical Diagnostics, Inc.)	High-Throughput CLIA	Spike	100%	100%	100%	100%
Elecsys Anti-SARS-CoV-2 (Roche)	High-Throughput ECLIA	Nucleocapsid	100%	99.8%	96.5%	100%
New York SARS-CoV Microsphere Immunoassay for Antibody Detection (Wadsworth Center, New York State Department of Health)	MIA	Nucleocapsid	88%	98.8%	79.4	99.4%

**Table 3:** Summary of most recent Emergency Use Authorization (EUA) issued antigen tests for SARS-CoV-2. All the information is collected from the Food, Drug and Administration (FDA) website.

Organization	Test Name	Test Result Time	Specimen Type	LOD	Characteristics
Access Bio, Inc.	CareStart COVID-19 Antigen test	10 min	Direct Nasopharyngeal Swab	8 × 10^2^ TCID_50_/mL	SARS-CoV-2 Nucleocapsid Protein-Specific, Lateral Flow, Visual Readout, Lab-based, Point-of-Care Testing (POC) but requires a sample preparation step and a trained operator to perform the test.
Quidel Corporation	Sofia 2 Flu + SARS Antigen FIA	15 min	Direct Nasal or Nasopharyngeal Swabs	4.17 × 10^5^ TCID_50_/mL	Mulitiplex Detection (SARS-CoV-2, Influenza A Virus, and Influenza B Virus), Nucleocapsid Protein-Specific, SARS-CoV, and SARS-CoV-2 Detection but cannot differentiate between them, Lateral Flow, Instrument-based Immunofluorescence Read, Lab-based, POC Testing but is limited to Sofia 2 Instrument for results, Trained Operator required.
Abbott Diagnostics Scarborough, Inc.	BinaxNOW COVID-19 Ag Card	15 min	Direct Nasal Swab	22.5 TCID_50_/swab	Singleplex SARS-CoV-2 Detection, No Specific to SARS-CoV-2 Detection alone (SARS-CoV Cross-reaction), Nucleocapsid Protein-Specific, Lateral Flow Immunoassay, Visual Colorimetric Pink/Purple Read but results interpretation is limited to individuals with color-impaired vision, POCT Testing but testing performance is limited by following meticulous testing instructions.
Lumira Dx UK Ltd.	Lumira Dx SARS-CoV-2 Ag Test	12 min	Direct Nasal Swab	32 TCID_50_/mL	Single-Use Microfluidic Fluorescence Immunoassay, SARS-CoV-2 Nucleocapsid Protein-Specific, Digital Instrument Read, Small Sample Testing Volume (one drop), POC Testing but limited to Healthcare Professional-Use-Only proficient in performing tests using the Lumira Dx Platform, Requires Lumira Dx Test Strip.
Becton, Dickinson, and Company (BD)	BD Veritor System for Rapid SARS-CoV-2 Detection	15 min	Direct Nasal Swab	1.4 × 10^2^ TCID_50_/mL	Chromatographic Immunoassay, SARS-CoV-2 Nucleocapsid Antigen-Specific, Digital Instrument Reading, Result Documentation Capabilities, Good Analytical Sensitivity, Laboratory-based, POC Testing but is limited to patient testing environments where test interpretation can only be done with the BD Veritor Plus Analyzer Instrument.
Quidel Corporation	Sofia SARS Antigen FIA	16 min	Direct Nasal or Nasopharyngeal Swabs	1.13 × 10^2^ TCID_50_/mL	Immunofluorescent Sandwich Assay, Lateral Flow, Singleplex Detection, SARS-CoV or SARS-CoV-2 Nucleocapsid Antigen-Specific but does not differentiate between them, Instrument Reading, Good Analytical Sensitivity, Results Affected by High Viscous Samples, Result interpretation limited to trained clinical laboratory personnel proficient in performing tests using Sofia and Sofia 2 instruments.
